# Prevalence of obstructive sleep apnea in Asian adults: a systematic review of the literature

**DOI:** 10.1186/1471-2466-13-10

**Published:** 2013-02-23

**Authors:** Aibek E Mirrakhimov, Talant Sooronbaev, Erkin M Mirrakhimov

**Affiliations:** 1 Kyrgyz State Medical Academy named after I.K. Akhunbaev, Akhunbaev Street 92, Bishkek, 720020, Kyrgyzstan; 2 National Centre of Cardiology and Internal Medicine named after М. Mirrakhimov, T.Moldo Street 3, Bishkek, 720040, Kyrgyzstan

**Keywords:** Obstructive sleep apnea, Obstructive sleep apnea syndrome, Epidemiology, Prevalence, Asia, Systematic review

## Abstract

**Background:**

Obstructive sleep apnea (OSA) is a common disease, affecting approximately 2% of women and 4% of men residing in Western communities. No systematically reviewed data are available about the prevalence of this disease in Asia, the most heavily populated continent.

**Methods:**

PubMed/Medline, Scopus and Google Scholar were searched for articles published from 1993 to May 2012 that reported the prevalence of OSA diagnosed via sleep monitoring and the prevalence of patients at risk for OSA as assessed by symptomatology and/or sleep questionnaires. We have also searched abstract database of major pulmonary and sleep scientific societies for relevant abstracts presented from 2010 to 2012. The following inclusion criteria were used: articles published in English, age ≥ 18 years, ≥ 100 participants in studies using sleep monitoring for the diagnosis of OSA, ≥ 300 participants in studies using questionnaires to detect patients at high risk for OSA. Exclusion criteria: duplicate publications, studies reporting the prevalence of central sleep apnea only, hospital based studies as well as studies assessing OSA prevalence among patients with resistant arterial hypertension, chronic kidney disease, heart failure and in patients with concomitant neurological disease.

**Results:**

Twenty four articles were found to meet the inclusion criteria, covering 47,957 subjects (26,042 men and 21,915 women) and four relevant abstracts were noted. OSA prevalence ranged from 3.7% to 97.3%. Male gender, older age, a higher BMI and waist to hip ratio, greater neck circumference, arterial hypertension, smoking, snoring and daytime sleepiness were associated with OSA. Sample size, difference between the populations studied and the fact that some works included patients with a high pre-test probability of OSA explain the difference in prevalence rates.

**Conclusion:**

This systematic review highlights the lack of data regarding the prevalence of OSA in Asians. Only a few studies provide an approximate estimate of the OSA burden in some Asian communities.

## Background

Obstructive sleep apnea (OSA) is a common medical condition and a form of sleep disordered breathing (SDB), which is characterized by repetitive complete and/or partial collapses (apnea and hypopnea respectively) of the upper airways. The disease is classified as mild, moderate and severe based on the number of apneas and/or hypopneas per hour of sleep, known as the apnea-hypopnea index (AHI). This is assessed by polysomnography (PSG) or other forms of sleep monitoring [1]. Obesity, aging, male sex, smoking and alcohol intake are the risk factors for OSA.

Subjects with OSA may complain of excessive daytime sleepiness (EDS) or insomnia, nocturia and morning headaches, but some patients with OSA may be asymptomatic. Several clinically helpful questionnaires are available for detecting patients at high risk for OSA such as the Berlin Questionnaire [2] and Epworth Sleepiness Scale (ESS) [3], but the diagnosis is made by PSG or by home sleep monitoring, and these questionnaires are only of ancillary use. For more general information on OSA, please refer to a well- written review article on this topic [4].

Asia is the most heavily populated continent, with some groups living in an underdeveloped environment. To date there are no published scientific reports on the general prevalence of OSA in Asia.

The rationale for this manuscript is to systemize the current data on the prevalence of OSA and patients at risk for OSA obtained from the Asian population and to highlight potential shortcomings, which should be addressed in future epidemiologic works.

## Methods

### Search strategy and selection criteria

The systematic review was performed according to the key tenets of the PRISMA guidance for systematic reviews [5]. PubMed/Medline, Scopus, Google Scholar databases were searched for articles published from 1993 to September 2012 that reported the prevalence of OSA diagnosed via instrumental sleep monitoring and full PSG and the prevalence of patients at risk for OSA among Asians as assessed by symptomatology (snoring, daytime sleepiness etc.) and/or questionnaires.

Scientific abstracts presented on the last three scientific meetings (2010–2012) of American Academy of Sleep Medicine, American Thoracic Society, American College of Chest Physicians, European Respiratory Society, Asian Pacific Society of Respirology were searched. The data from the relevant abstracts will be discussed in the discussion only, due to limited amount of information provided in them.

The search terms were: obstructive sleep apnea, obstructive sleep apnea syndrome, sleep disordered breathing, prevalence, risk factors for obstructive sleep apnea, Asia and the names of Asian countries (including Israel and Turkey) as well as a combination of these. Reference lists of relevant articles were checked to find potentially relevant publications that may have been overlooked by the electronic search.

The articles identified, were screened by title and abstract, and selected for full text review if they met the following inclusion criteria: studies performed in Asia, documenting OSA prevalence and/or prevalence of patients at risk for OSA, articles in English, age ≥ 18 years, ≥ 100 participants in studies using PSG or other forms of instrumental sleep monitoring for the diagnosis of OSA, ≥ 300 participants in studies not using PSG for OSA detection. Even, if the study’s goal was not to assess the epidemiology of OSA, but the prevalence was examined and included in the results, such studies were eligible for inclusion.

Specific exclusion criteria: duplicate publications, studies reporting the prevalence of central sleep apnea only, hospital based studies as well as studies assessing OSA prevalence among patients with resistant arterial hypertension, chronic kidney disease, heart failure and stroke/neurological disease, since the prevalence of OSA is much higher in these groups than in the general population [6-8].

### Outcomes studied

The outcomes were the prevalence of OSA diagnosed by full PSG and other types of instrumental sleep monitoring. Another task was to study the prevalence of patients at risk for OSA as assessed by symptomatology and questionnaire use.

### Data extraction and quality assessment

General data extraction included the information on the publication dates, methodology of the study, number of individuals enrolled and their characteristics as well as definition of OSA and its measurement (symptomatology/questionnaire vs. instrumental sleep monitoring).

### The quality assessment tool was modified from the checklist recommended by the centre for

Reviews and Dissemination, York, United Kingdom and included [9]: clear goals and objectives of the study, clear and appropriate methods, risk of bias in selection, risk of bias in study outcomes (0 points for obvious bias risk present, 1point if no apparent bias risk present), diagnosis of OSA (0 points for non-instrumental sleep study, 1 point for instrumental sleep monitoring other than PSG, 2 points for PSG), discussion of the limitations, funding information and conflicts of interest. Since, all suitable studies were cross-sectional works; therefore, prospective methodology was not scored. The maximal score for an individual study in terms of quality was 8 points. The maximal quality score for studies using questionnaires was 6, given that they did not use instrumental sleep monitoring.

## Results

A total 732 articles were found on the prevalence of OSA in Asia and the prevalence of patients at risk for OSA based on questionnaire and/or symptom assessment. Of these 676 articles were excluded after abstract screening for the following reasons: not focused on the studied outcomes (n-416), studies in pediatric population (n-90), reviews, comments/editorials (n-67), articles not published in English (n-39), small sample (n-33), studies performed in patients with certain comorbidities (n-20), studies performed outside Asia (n-9),  animal studies (n-7).

Fifty one full text articles were evaluated, with 28 articles excluded for the following reasons: hospital- based studies (n-18), not focused on studied outcomes (n-7), duplicate publications (n-2). Twenty four articles covering 47,957 patients (26,042 men and 21,915 women) were eligible based on the aforementioned criteria. Many studies were excluded for more than one reason.

**Figure 1:**
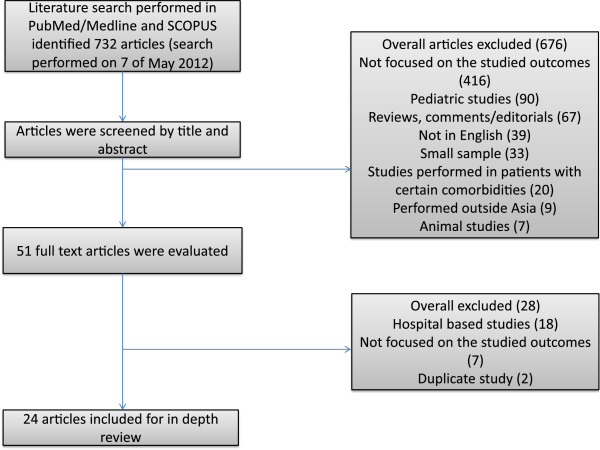
Flow chart of selected studies.

Below, we review the data on the prevalence of patients at risk for OSA first, and then the data on the prevalence of OSA based on the instrumental assessment. The diagram of the literature search is shown in Figure 1.

### Community studies using sleep questionnaires

Ten manuscripts were community studies. These included 32,508 participants (17,580 women and 14,928 men): two studies from Turkey (n-6,117) [10,11], two studies from Taiwan/China (n-5,263) [12,13], two studies from Iran (n-4,056) [14,15], one study each from Japan (n-8,483) [16], Thailand (n-4,680) [17], Singapore (n-2,298) [18] and Malaysia (n-1,611) [18]. A summary of the studies is presented in Table 1.

**Table 1:** Summary of the community studies without PSG assessment

**Study**	**Country**	**Study design**	**Population studied**	**Prevalence of patients at high risk for OSA**	**Risk factors**	**Quality assessment score**
Ozdemir et al. [10]; 2005	Turkey	Cross-Sectional study using Sleep and Health questionnaire	n-5,339 females-50.6% mean age- 38.4 years BMI-no data provided	40.3% had insomnia, 37% were snorers, 24% had EDS and 6.4% had episodes of witnessed apneas	None reported.	2 points: single area study, no discussion of the potential limitations and no funding information.
Kart et al. [11]; 2010	Turkey	Cross-Sectional study using adapted version of the Berlin questionnaire	n-778 males-100% coal miners- 54.3% mean age-43.3 years for coal miners and 44.3 years for controls mean BMI-27.2 kg/m^2^ and 26.1 kg/m^2^ for coal miners and controls respectively	40.7% of all included participants had snoring, 4.37% had witnessed apneas and 3.85% had EDS.	None reported.	3 points: single area study, no funding information.
Liu et al. [12]; 2004	Taiwan/China	Cross-Sectional study using modified questionnaire via telephone interviewing	n-1,252 females-51.6% mean age 38.6 years BMI-data no provided	46.8% were snorers	Male gender and age range of 40–59 years	2 points: single area study, no discussion of the potential limitations and no funding information.
Chuang et al. [13]; 2008	Taiwan/China	Cross-sectional study using modified questionnaire via telephone interviewing	n-4,011 females-59.2% age-15-30-15.8%; age-30-50-36.1%; age > 50-48.1% mean BMI-23,15 kg/m^2^	Overall presence of snoring was 59.1%: 60.8% for males and 42.5% for females Snoring in females <50 years was present among 37.1% vs. 49.1% in females > 50 years. Snoring in males < 50 years was present among 57.9% vs. 63% in males > 50 years. Overall presence of witnessed apnea was 2.6%: 3.4% for males and 1.9% for females. Witnessed apnea in males < 50 years was present among 2.3% vs. 5.3% in males > 50 years. Witnessed apnea in females < 50 years was present among 1.4% vs. 2.8% in females > 50 years	Male gender and age > 50 years	4 points: no funding information provided.
Amra et al. [14]; 2011	Iran	Cross-Sectional study using Berlin questionnaire	n-3,529 females-53.3% mean age-40.47 and 38.61 years for males and females respectively mean BMI-25.05 and 26.39 kg/m^2^ for males and females respectively	4.98% of participants were at high risk for OSA (102 females and 74 males)	Obesity, older age and illiteracy	3 points: single area study, no discussion of the potential limitations.
Khazaie et al. [15]; 2011	Iran	Cross-Sectional study using Berlin questionnaire	n-527 males-77.9% mean age-48.6 years mean BMI-25.1 kg/m^2^	27.3% were at high risk for OSA. 9.67% reported witnessed apneas	Illiteracy	3 points: single area study, no funding information.
Nagayoshi et al. [16]; 2011	Japan	Cross-Sectional study using modified questionnaire	n-8,483 females-63% mean age-59.8 years mean BMI- 23.4 kg/m^2^	24% of males and 10% of females had snoring.	Alcohol intake and possibly smoking	6 points
Suwanprathes et al. [17]; 2010	Thailand	Cross-Sectional study using modified questionnaire	n-4,680 females-54% median age- 34 years mean BMI- 26.03 kg/m^2^ for patients with sleep complaints and 22.77 kg/m^2^ for others	4.6% of general sample had snoring and EDS. 5.3% of males and 3.5 of females had snoring.	Male gender, greater waist circumference and BMI	4 points: single area study, no discussion of the potential limitations.
Khoo et al. [18]; 2004	Singapore	Cross-Sectional study using modified questionnaire	n-2,298 (35%-Chinese, Malay-33.7% and Indians-31.3%) females-52% age 20–59 years-85.4% and age > 60 years-14.6% BMI ≥ 30 kg/m^2^ -6.2%	6.8% of the total population complained of snoring	Male gender, age > 60 years, Indian ethnicity, family history, obesity, neck circumference > 40 cm and smoking	6 points
Kamil et al. [19]; 2007	Malaysia	Cross-Sectional study using modified questionnaire and Epworth Sleepiness Scale for EDS	n-1,611 (47.1%-Malay, 36.6%-Chinese, 11.3%-Indian and 5%-others) males-52.9% mean age-49 years BMI-data not provided	47.3% had snoring, 15.2% had witnessed apneas and 14.8% had EDS	Older age, Chinese/Indian origin vs. Malay, smoking (both past and current), obesity and sedative use	4 points: no discussion of the potential limitations and no funding information.

Included studies used the Berlin questionnaire, Sleep and Health questionnaire, ESS and modified sleep questionnaires. The prevalence of patients at high risk for OSA ranged from 4.98% [14] to 27.3% [15], both from Iranian studies. This difference could be explained by the fact that the study with a lower prevalence [14] included a higher number of women and a much younger population, as well as a larger sample.

Other studies reported the prevalence of snoring, witnessed apnea, EDS and insomnia. The lowest snoring prevalence of 4.6% was reported in Thailand [17] and the highest prevalence of 59.1% was in Taiwan [13]. The study from Thailand [17] included a much higher number of women and the population was younger than in the study from Taiwan/China [13]. The lowest prevalence of witnessed apneas was 2.6% in Taiwan [13], and the highest was 15.2% in Malaysia [19]. Symptoms of EDS ranged from 3.85% [11] to 24% [10], both in Turkish studies. The difference in EDS prevalence could be explained by the different sample sizes and populations studied.

Male gender, older age, greater BMI and neck circumference, smoking, alcohol intake, sedative use, health illiteracy and Indian and Chinese ethnicity were related to a high risk for OSA.

As already mentioned the questionnaires have some limitations and are only suitable for the detection of patients with a high pretest probability of OSA. The included studies were of different methodological quality, and some did not report such valuable information as BMI. Some studies reported data only in terms of symptomatology. Despite having, a good sensitivity for OSA, the specificity and positive predictive value for the disease are much lower than desired, and these factors interfere with the translation of the results of OSA questionnaires into real life scenario. The quality scores of these articles are presented in Table 1.

### Community studies with instrumental sleep monitoring and/or full PSG assessment

Fourteen studies were community based studies using instrumental sleep monitoring and/or full PSG including 15,449 patients (11,114 men and 4,335 women). There were five Chinese studies (n-6,447): three from Hong-Kong (n-3,145) [20-22], two studies from mainland China (3,302) [23,24]; four Japanese studies (n-3,475) [25-28], two Indian studies (n-4,665) [29,30], and one each from Korea (n-5,020) [31], Malaysia (n-279) [32] and Singapore (n-106) [33]. The summary of the studies is presented in Table 2.

**Table 2:** Summary of the community studies with instrumental sleep and/or full PSG assessment

**Study**	**Country**	**Study design**	**Population studied**	**OSA/SDB prevalence**	**Risk factors and associated states**	**Quality assessment score**
Ip et al. [20]; 2001	China/Hong-Kong	Cross-Sectional study using sleep questionnaire and PSG	n-784 (153 underwent PSG) Males-100% Mean age-41.2 years Mean BMI-23.9 kg/m^2^	23% had snoring. 41.8% were diagnosed with OSA by PSG. Estimated OSA and OSAS prevalence was 8.8% and 4.1%	Older age, higher BMI, snoring and time taken to fall asleep	6 points: single area study, no funding information.
Ip et al. [21]; 2004	China/Hong-Kong	Cross-Sectional study using sleep questionnaire and PSG	n-884 (105 underwent PSG) Females-100% Mean age-41.6 years Mean BMI-22.4 kg/m^2^	15% had snoring. AHI ≥ 5, AHI ≥ 10 and AHI ≥ 15 were present among 30%, 15% and 10% respectively. Estimated prevalence of OSA and OSAS was 3.7% and 2.1% respectively	Older age and higher BMI	5 points: single area study, no discussion of potential limitations and no funding information.
Hui et al. [22]; 2006	China/Hong-Kong	Cross-Sectional study using sleep questionnaire, 4 channel home sleep monitoring and PSG	n-1,477 (211 undergone 4 channel home sleep study and 25 have been assessed by PSG) Males-65.74% Mean age-45.3 years Mean BMI- 24.9 kg/m^2^	EDS was present in 60.9%, falling asleep in 24%, snoring in 23.9% and witnessed apnea in 3.7%. RDI ≥ 5 was present in 83.9% and RDI ≥ 15 was present in 17.5%. It was estimated that OSA and OSAS was present in 8.4% and 4.4% respectively	Higher BMI, neck circumference and snoring	6 points: single area study, no funding information.
He et al. [23]; 2010	China	Cross-Sectional study using PSG	n-2,297 Males-86.24% Mean age-46 years Mean BMI- 27.64 kg/m^2^	88.81% were diagnosed with OSA and 51.28% were diagnosed with severe OSA	Systolic and diastolic blood pressure	6 points: no discussion of the potential limitations and no funding information.
Chen et al. [24]; 2011	China	Cross-Sectional study using PSG	n-1,035 Males-83.67% Mean age-45 years Mean BMI- 26.2 kg/m^2^ in mild OSAS, 27.5 kg/m^2^ for moderate and 28.8 kg/m^2^ for severe OSAS	75.9% had OSAS and 37.7% had AHI > 40.	ESS score correlated with ODI, AHI and BMI. ODI was associated with ESS score.	6 points: Single area study and no discussion of the potential limitations.
Tanigawa et al. [25]; 2004	Japan	Cross-Sectional study using pulse oximetry monitoring	n-1,424 Males-100% mean age-58.6 years Mean BMI-24 kg/m^2^	31.46% and 9% had ODI of 5–15 and ODI > 15 respectively	Arterial hypertension, BMI, smoking and older age	4 points: single area study, PSG was not used, no discussion of the potential limitations and no funding information.
Cui et al. [26]; 2006	Japan	Cross-Sectional study using pulse oximetry monitoring	n-1,313 Males-96% Mean age- for 3% ODI of 5–14 was 47 years and 47.3 years for 3% ODI > 15Mean BMI- 3% ODI of 5–14 was 25.6 kg/m^2^ and 28.5 kg/m^2^ for 3% ODI > 15	Subjects aged 40–69 have SDB prevalence of 8.5% compared to 4% among aged 20-39	age ≥ 40 years, higher BMI, ESS > 11 and arterial hypertension	6 points: PSG was not used, no funding information.
Okabayashi et al. [27]; 2007	Japan	Cross-Sectional study using home pulse oximetry and then PSG study among Japanese workers	n-368 underwent home pulse oximetry and 153 were screened with PSG Males-100% Mean age-45.7 years Mean BMI-25.8 kg/m^2^	313 patients out of 368 (85%) had ODI ≥ 5 events. 149 out of 153 (97.3%) had OSA		7 points: no funding information.
Asaoka et al. [28]; 2010	Japan	Cross-Sectional study using 3 channel home sleep monitoring in patients with positive ESS and/or snoring, witnessed apneas, BMI ≥ 25 kg/m^2^ and concomitant arterial hypertension	n-370 undergone home sleep study; 129 participants undergone PSG study Males-100% Mean age-44.5 years Mean BMI-24.3 kg/m^2^	OSA was present among 3.7%		5 points: single area study, PSG was not used, no funding information
Sharma et al. [29]; 2006	India	Cross-Sectional study using sleep questionnaire and PSG	n-2,150 Males-52.8% Mean age-43.9 years for snorers Mean BMI- 27 kg/m^2^ 151 subjects (77 snorers and 74 nonsnorers) undergone PSG	25.16% subjects were found to have OSA and 7.28% subjects were found to have OSAS (among who underwent PSG). It was estimated that 13.74% and 3.57% of the population should have OSA and OSAS respectively	Male gender, older age, snoring, higher BMI and greater waist to hip ratio	6 points: single area study, no funding information.
Reddy et al. [30]; 2009	India	Cross-Sectional study using sleep questionnaire and PSG	n-2,505 Males-50.4% Mean age-41 years Mean BMI-24.3 kg/m^2^ 360 subjects (287 snorers and 73 nonsnorers) undergone PSG	26.94% subjects had OSA and 12.2% subjects had OSAS (among who underwent PSG).	Male gender, BMI ≥ 25 kg/m^2^ and abdominal obesity	7 points: single area strudy.
Kim et al. [31]; 2004	Korea	Cross-Sectional study using home sleep study or PSG	n- 457 Males- 67.6% Mean age-49.1 years for males with snoring and 54.3 years for females with snoring Mean BMI-26.5 kg/m^2^ for males with snoring and 26.6 kg/m^2^ for females with snoring	OSA and was found in 42% men and 20% women and OSAS was present in 4.5% men and 3.2% women	Male gender, higher BMI and hypertension	7 points: single area study.
Yusoff et al. [32]; 2010	Malaysia	Cross-Sectional study using PSG	n-279 Males-100% Mean age-43.8 years Mean BMI- 29.4 kg/m^2^ for patients with OSA	44.3% had AHI ≥ 5 and 6.6% had xAHI ≥ 30.	Older age, greater BMI and neck circumference, snoring and hypertension	7 points: no discussion of the potential limitations.
Puvanendran et al. [33]; 1999	Singapore	Cross-Sectional study using PSG	n-106 male: female ratio 9:1 No data on mean age and mean BMI are provided	87.5% had OSA and 72% had OSAS. It was calculated that 15.7% of the Singaporean population may have OSA.		6 points: no discussion of the potential limitations and no funding information.

Six studies used PSG [20,21,23,24,32,33], 2 studies used PSG and a sleep questionnaire [29,30], one study used a sleep questionnaire, home sleep monitoring and PSG [22], one study used home sleep study and PSG [31], one study used overnight pulse oximetry monitoring and PSG [27], one study used three channel home sleep monitoring [28] and two studies used overnight pulse oximetry [25,26]. The smallest sample consisted of 106 participants (Singaporean study) [33], and the largest consisted of 2,505 participants (Indian study) [30]. Men were predominant studied group in most studies, whereas one study enrolled exclusively women [21].

The mean age of participants ranged from 41.2 years in the study from Hong-Kong/China [20] to 58.6 years in the Japanese study [25]. The mean BMI varied from 22.4 kg/m^2^ in the study from Hong-Kong/China [21] to 29.4 kg/m^2^ in the Malaysian study [32]. The study from Singapore did not report data on either mean age or mean BMI [33].

OSA prevalence ranged from 3.7% one Japanese study [28] to 97.3% in another study from Japan [27]. This huge difference may be attributed to the different populations studied with a greater BMI and older age in the latter study. Another potential explanation is that the patients that underwent PSG, in the study by Okabayshi et al. [27] had a remarkably high pre-test probability of OSA, since they had been tested via home pulse oximetry.

Some studies reported the prevalence of OSA with daytime symptoms, which ranged from 3.2% in women and 4.5% in men in the Korean study [31] to 72% in the study from Singapore [33]. However, the difference in the sample size should be kept in mind, along with the fact that the Singaporean study did not provide data on the mean age or mean BMI. Furthermore, it was impossible to extract the original article, since the journal in which it was published no longer exists.

Some studies reported the estimated OSA/OSAS prevalence [20-22,29], which ranged from 3.7%/2.1% in the study performed by Hong-Kong researchers in women [20] to 13.74%/3.57% in the Indian study [29]. The Indian study [29] had a predominantly male sample, and the population was heavier and older than in the study from Hong-Kong [20], which recruited exclusively women and these factors can help explain the difference in estimated disease burden.

Male gender, older age, greater BMI, neck circumference and waist to hip ratio, increased blood pressure, smoking, snoring, time taken to fall asleep and a higher ESS score were associated with OSA in the aforementioned studies.

Since the studies were of different methodological quality, tested different populations, and used various types of sleep monitoring to assess OSA and since many countries lack any epidemiologic data, it is particularly difficult to extrapolate the data to the global disease prevalence in Asia. However, the studies performed by Ip et al. [20,21], Hui et al. [23] and Reddy et al. [30] are likely to be representative of their respective populations. Based on these results it is likely that in Hong-Kong study the average prevalence of OSA is around 7%, and that of OSA with daytime symptoms is around 3.5%, whereas in India the prevalence is 13.74% for OSA and 3.57% for OSA with daytime symptoms.

## Discussion

OSA is a particularly common and underrecognized medical disorder. It is associated with increased morbidity and mortality from cardiovascular causes, and traumatic accidents due to EDS. OSAS, which is characterized by abnormal AHI and symptoms of EDS, is present in 2% of women and 4% of men living in Western communities.

The Asian continent is heavily populated, and many groups live in an underdeveloped environment. These factors pose some difficulties in assessing the disease burden in this area. As expected most studies came from developed countries such as Japan, China, Singapore, Turkey and others. Interestingly, no epidemiologic studies have been conducted in Israel, despite the many works performed there in the area of OSA pathophysiology.

The retrieved studies were divided into two main groups: studies using sleep questionnaires and those studies using various sleep studies including PSG. Community studies are more likely to portray epidemiology with better accuracy than single center hospital studies. Second, hospital studies usually enroll patients with a high pre-test probability of diagnosis, which is true for studies using questionnaires/symptomatology as well as PSG studies. Given the latter concern, hospital based studies were excluded from this review.

It is well known that sleep questionnaires despite being useful in assessing risk for OSA, are not interchangeable with instrumental sleep studies and cannot quantify the severity of disease. Thus, the prevalence of people at high risk for OSA based on questionnaires cannot be simply converted into the prevalence of OSA. Another flaw is that questionnaires such as the ESS cannot rule out other sleep disorders; in fact this scale was primarily invented to detect EDS [3], and not OSA or patients at high risk for OSA.

Ten community studies that used sleep questionnaires and assessed OSA associated symptoms were found. Most of these studies used modified versions of sleep questionnaires with questions regarding snoring, nocturnal apneas, EDS, daytime fatigue etc. The smallest sample was 527 in the Iranian study [15] and the largest sample was 8,483 in the Japanese study [16]. The reported prevalence of risk for OSA prevalence ranged from 4.98% to 27.3%, both in the Iranian studies [14,15]. Male gender, older age, higher BMI, greater waist to hip ratio and neck circumference, illiteracy, alcohol intake and smoking were associated with at high risk for OSA.

Fourteen community studies using sleep monitoring were found. Some of these were two phase studies that used a sleep questionnaire and sleep monitoring. The smallest sample was 106 in the study from Singapore [33] and the largest sample was 5,020 in the Korean study [31]. The prevalence of OSA ranged from 3.7% in the Japanese study [28] to 88.81% in the Chinese study [23]. Male gender, older age, a higher BMI and waist to hip ratio, greater neck circumference, arterial hypertension, smoking, snoring and a higher ESS score were related to OSA. The striking difference in prevalence can be attributed to variations in sample size and different populations studied, since some predominantly assessed patients with OSA related symptoms such as snoring, witnessed apnea etc.

Since the studies were of different methodological quality, tested different populations, used various types of sleep monitoring to assess OSA and many countries lack any epidemiologic data, it is particularly difficult to extrapolate the data to the global OSA/OSAS prevalence in Asia. However, the studies performed by Ip et al. [20,21], Hui et al. [22] and Reddy et al. [30] are likely to be representative of their studied populations. Based on these results it is likely that average prevalence of OSA is around 7%, and OSAS prevalence is around 3.5% in Hong- Kong and 13.74% for OSA and 3.57% for OSAS in India.

Several abstracts from the databases mentioned in the “Search strategy and selection criteria” were found relevant for discussion. We used the same sample size cut off for abstract reports as for regular articles. However, it is necessary to note that it is difficult to analyze the abstracts in a thorough fashion due to limited word count. Pablo et al. screened 458 Philippine medical students with Berlin questionnaire and ESS [34]. These researchers showed that approximately 75.9% of studied participants had symptoms of EDS, but no data was provided on the Berlin questionnaire scores. It is essential to mention that their sample of medical students may not represent a general Philippine population. Liu et al. interviewed 666 patients undergoing anesthesia with Berlin questionnaire at West China Hospital of Sichuan University, China [35]. These investigators found that 11.7% were found to be at high risk for OSA. The individuals at high risk for OSA had a greater prevalence of high blood pressure, snoring and EDS. Ardic et al. screened 5,021 adults (2,598 women) with Berlin questionnaire in Ankara, Turkey [36], abstract 0418. These researchers showed that 13.7% of the screened population was at high risk for OSA. Li et al. retrospectively analyzed the PSG data of 2,335 individuals (1,960 men) who were suspected to have OSA [36], abstract 0466 in West China Hospital of Sichuan University, China. They showed a greater prevalence of OSA among men. Since, the above study was assessing the PSG of the patients who likely were initially at high risk for OSA, it is impossible to extrapolate this data to general Chinese population. Chang et al. studied 284 subjects with snoring problems with home sleep monitoring [36], abstract 0467. These investigators found that 61.3% of individuals had SDB. Since, these individuals had complaints of snoring before the actual sleep testing; they were at high risk for OSA, which make this finding inapplicable to general Chinese population.

Overall, based on the present studies it is difficult to estimate the potential prevalence of OSA/OSAS in the general population in these countries, because of the heterogeneity of the subjects and methods used to assess patients at risk and different types of sleep monitoring devices used.

Moreover, there are still no prevalence data for most Asian countries, including Bangladesh, Mongolia, Syria, former Soviet Union countries located in Asia and others. Future studies should recruit patients, who are generally considered to be at low risk for OSA to give a better understanding of the OSA burden on the Asian continent. This will give a better insight into the prevalence of OSA in the general Asian population. Therefore, more studies are needed to provide a better knowledge on the OSA burden in the Asian continent.

### Limitations

This systematic review has some potential limitations: first, only articles written in the English language were included. Therefore, some relevant studies may have been missed. Another potential drawback could arise from be due to limitations of the search engines used, such as PubMed/Medline, Scopus, Google Scholar and abstracts of the American Thoracic Society, American College of Chest Physicians, American Academy of Sleep Medicine, European Respiratory Society and Asian Pacific Society of Respirology.

## Conclusion

Many published studies assessed people with high pre-test probability of OSA; thus their results may overestimate the true burden of disease. Therefore, more studies are needed to improve our knowledge on the OSA burden in Asia.

## Abbreviations

AHI: Apnea-hypopnea index; BMI: Body mass index; EDS: Excessive daytime sleepiness; ESS: Epworth sleepiness scale; OSA: Obstructive sleep apnea; OSAS: Obstructive sleep apnea syndrome; PSG: Polysomnography; SDB: Sleep disordered breathing.

## Competing interest

The authors declared that they have no competing interest.

## Authors’ contribution

All authors read and approved the final manuscript.

## Pre-publication history

The pre-publication history for this paper can be accessed here:

http://www.biomedcentral.com/1471-2466/13/10/prepub
